# The short-term effects of COVID-19 on HIV and AIDS control efforts among female sex workers in Indonesia

**DOI:** 10.1186/s12905-021-01583-z

**Published:** 2022-01-26

**Authors:** Robert J. Magnani, Dewa Nyoman Wirawan, Anak Agung Sagung Sawitri, I. Gusti Agung Agus Mahendra, Dewi Susanti, Ni Kadek Ayu Dwi Utami Ds, Dedison Asanab, Jessie Olivia Yunus, Cahyo Heri Setiabudi, Adi Nugroho, Asti Setiawati Widihastuti, Oldri Sherli Mukuan, Pande Putu Januraga

**Affiliations:** 1 Faculty of Public Health, University of Indonesia, Jakarta, Indonesia; 2 Kerti Praja Foundation, Denpasar, Bali, Indonesia; 3 Department of Community and Preventive Medicine, Faculty of Medicine, Udayana University, Denpasar, Bali, Indonesia; 4 Center for Public Health Innovation, Faculty of Medicine, Udayana University, Denpasar, Bali, Indonesia; 5 United Nations Population Fund, Jakarta, Indonesia; 6 School of Public Health, Faculty of Medicine, University of Lambung Mangkurat, Banjarbaru, South Kalimantan Indonesia

**Keywords:** Indonesia, COVID-19, HIV/AIDS program impact

## Abstract

**Background:**

The COVID-19 pandemic has raised concerns as to its impact on other health programs. One program that appears particularly vulnerable is HIV and AIDS. We undertook an assessment of COVID-19 impact on HIV control efforts in Indonesia for a sub-population that has received little attention in the global literature—female sex workers (FSW).

**Methods:**

The study was undertaken in 23 National AIDS program priority districts. Four sources of monthly data during January–July 2020 were considered. COVID-19 infection data were extracted from national and district surveillance systems. Combination prevention program outputs were reported by civil society organizations (CSOs) providing community support services to FSW. These organizations also undertook monthly scans of levels of commercial sex activity and HIV testing availability. We also considered data from an ongoing HIV community screening trial. The primary mode of analysis entailed comparisons of levels and trends of indicators from the four data series.

**Results:**

Commercial sex activity was severely curtailed in April–May in many districts. While recovering to pre-COVID-19 levels in “Localization” areas, the number of active FSW in July was one-third below that in February. HIV testing service availability declined by 50% at health facilities before recovering slowly, while mobile clinic services largely ceased during April–June. Numbers of FSW reached, condoms distributed, FSW tested for HIV, HIV cases detected, and FSW starting treatment all declined precipitously in April/May but had largely recovered to pre-COVID-19 levels by July. We found only a temporary dip in treatment initiation rates among HIV positive FSW and no discernible impact on treatment retention. The HIV community screening trial data revealed significant demand for HIV testing among FSW that was not being met even before the onset of COVID-19.

**Conclusions:**

COVID-19 has had at least short-run economic effects on FSW and the national response to HIV and AIDS targeting FSW. However, the effects appear to have been cushioned by community-based services and support in study districts. The findings make a compelling case for the expansion of community-based services irrespective of the future trajectory of COVID-19. As COVID-19 has not yet been contained, the trajectory of economic activity and service delivery is uncertain.

## Introduction

On 30 January 2020, the World Health Organization (WHO) designated the Novel Coronavirus (COVID-19) a public health emergency of international concern and on 11 March 2020 a pandemic [1]. By that time, more than 205,000 COVID 19 cases had been confirmed in 166 countries and territories, with more than 8648 deaths reported [2]. By 31 July 2020, the number of infections had risen to over 18 million and the number of deaths to nearly 690,000 [2].

As the COVID-19 pandemic storms the globe, governments are taking extraordinary measures to contain the virus and mitigate its health, social and economic impacts. Although blunting the pandemic clearly requires the highest immediate health sector attention, there is a clear danger that emergency epidemic responses will result in at least a short-term disruption in non-COVID-19 health programs and services and possibly medium-to longer-term collateral damage [3]. The situation is especially dire in low- and middle-income countries where under-developed public health and social support systems are being put under further stress [4].

Already experiencing declining international financing [5], one health program that appears to be particularly vulnerable is HIV and AIDS. Indeed, the WHO has voiced its concerns that access to HIV medicines is already being severely impacted by COVID-19 as the global HIV and AIDS response stalls [6] and pointed out the costs of inaction to mitigate the damage [7]. Several studies have attempted to project or anticipate the magnitude of this impact. One modelling study estimated in high burden settings HIV-related deaths over a 5-year period would increase by up to 10% compared to if there were no COVID-19 epidemic, with the greatest impact coming from interruption to ART during periods of high or extremely high health system demand [8]. Another study that reviewed the COVID-19 responses in sub-Saharan African countries concluded that such measures “might result in unprecedented difficulties among vulnerable segments of society to access essential services, including antiretroviral and prophylactic drugs among people living with HIV [9]. Adepoju [10] expresses similar concerns regarding impact on the responses to both HIV and TB in Nigeria. Other publications have assessed the potential impact on facility-based HIV testing in sub-Saharan Africa [11], the potential impact on food security among patients on treatment [12], the potential for physical distancing measures to increase the social isolation of people living with HIV and AIDS (PLHIV) [13], and the double burden of COVID-19 and HIV for migrant workers [14].

Several published studies have provided new empirical evidence on COVID-19 impact: Harkness et al. [15] report on the behavioral and psychosocial impact among Latino MSM in Miami, Florida, USA; Jiang et al. [16] on the impact of the pandemic on the availability and quality of HIV and AIDS-related health services in Wuhan, China; and Odalele et al. [17] on the impact of COVID-19 on HIV financing in Nigeria. In a survey of infectious disease physicians in 19 Eastern and Central European countries, Kowalska et al. [18] found that while no country reported HIV clinic closures, HIV clinics were operating normally in only six countries (31.6%). In 11 countries (57.9%) physicians were sharing HIV and COVID-19 care duties. A UNAIDS-led survey of more than 20,000 LGBTI people in 138 countries conducted in April and May 2020 found that three quarters (74%) were fully or partially locked down, leading to economic consequences for many [19]. Of concern, 21% of participants living with HIV reported that they had experienced “interrupted or restricted access” to refills of antiretroviral therapy drugs and 42% of those said they had less than a month’s supply on hand. Worrisome disruptions were also reported for pre-exposure prophylaxis and access to HIV testing. In a biweekly qualitative survey undertaken by The Global Fund for AIDS, TB and Malaria (GFATM) in more than 100 countries, results to date suggest that 85% of national HIV programmes have been disrupted, with 18% experiencing high or very high levels of disruption [20].

This article reports the results of an early assessment of the effects of COVID-19 on HIV and AIDS control efforts in Indonesia for a sub-population that has received little attention to date in the global COVID-19/HIV literature—female sex workers (FSW). FSW and their clients are priority or key populations for AIDS control in Indonesia. Recent estimates indicated that there were 271,624 FSW (95% uncertainty bounds 128,114–364,313) and 4,688,216 men (95% uncertainty bounds 1,386,104–6,180,385) that had availed of the services of FSW in the prior 12 months in the country in 2019 [21]. Epidemic modelling indicated that, combined, FSW and their clients accounted for 24% of new infections in 2019 and 14% of all PLHIV [22]. The latter figure understates the importance of heterosexual commercial sex-related transmission in driving numbers of PLHIV because it excludes former sex workers and men who had engaged with FSW in prior years. As in many settings, Indonesian FSW are vulnerable to HIV transmission due to their limited power to negotiate condom use in commercial sex transactions as well as gender-based violence. COVID-19 threatens to increase this vulnerability by reducing access to HIV and AIDS-related health services, including community outreach support and access to prevention commodities. In addition, reduced numbers of clients due to population mobility restrictions and “entertainment area” closures might compel women to be less insistent on condom use in order to seize whatever income-generating opportunities present themselves.

## Methods and materials

The study was undertaken in the 23 highest priority cities and districts for the Indonesian National HIV and AIDS Program. While comprising only 5% of cities/districts in the country, in 2019 these cities/districts contained an estimated 24% of all FSW, 41% of FSW reached via program outreach, and 37% of FSW tested for HIV [22]. The study was conducted in parallel with a community randomized controlled trial of community HIV screening among FSW in the 23 districts.

### Data

Four (4) sources of secondary data were used in the study. As the official government COVID-19 surveillance system [23] only reports data at the national and provincial levels, we extracted COVID-19 infection rate and population data from district surveillance systems, websites and other official communications. Monthly infection rates per 1000 population were calculated for each of the study districts. Links to the data sources used in the calculations are provided in the article References.

The second source of data was program data maintained by local implementing units (IUs) of a comprehensive national HIV and AIDS program for FSW being managed by the United Nations Population Fund (UNFPA) with technical support from the Indonesian National Association of Sex Workers (OPSI) on behalf of the Ministry of Health. The program provides comprehensive combination prevention services to FSW in a total of 88 cities and districts. IUs report monthly on numbers of FSW reached, condoms distributed, FSW tested for HIV and found to be HIV-positive, and FSW starting treatment and retained on treatment for 3 months.

Third, IU Program Coordinators and outreach workers completed monthly online surveys using standardized forms to produced estimates of numbers of “entertainment” venues that remained open, numbers of FSWs who were still working, numbers of clients, and numbers of public service delivery points that continued to provide HIV testing services either in health facilities or via mobile clinics. Data collection entailed making scans of the commercial sex environment and gathering data from District Health Offices.

Finally, data on numbers of FSW receiving community-based screening for HIV were gathered from records being maintained for an ongoing trial of community-based screening/self-testing intervention that was being implemented by the researchers for this article in 15 of the 23 cities and districts included in this study. The protocol for this study may be found in reference [24].

The first three sources of data were exempt from human subject’s ethical review as they consisted of aggregate data that contained no information of any kind on individuals. It was confirmed and decided by Institutional Review Board of Faculty of Medicine Udayana University/Sanglah Public Hospital, Denpasar, Bali. The fourth source of data underwent ethical review at the same IRB and was assigned approval number 612/UN 14.2.2.VII.14/LT/2020.

Informed consent was not required and was waived for the first three sources of data as confirmed by Institutional Review Board of Faculty of Medicine Udayana University/Sanglah Public Hospital, Denpasar, Bali. For the HIV community screening study, informed consent was obtained from all eligible study participants before they received HIV testing. The consenting procedure was conducted online. Participants were allowed to read consent form by themselves or be assisted by outreach workers. The consent form was also developed as a video for those who were not able or willing to read. Follow up questions were asked after study participants had read or viewed the consent form/video to ensure that they fully understand what they were consenting to.

### Variables and measures

The primary mode of analysis entailed the comparison of levels and trends of indicators from the four data series. We begin by documenting the evolution of COVID-19 in the 23 study districts. The number of COVID-19 cases diagnosed monthly in each district per 1000 population was the metric for this analysis. The statistical significance of the onserved trend was assesses via analysis of variance (ANOVA). We then consider trends in the functioning of the commercial sex industry and availability of HIVtesting services in these districts as context for the analyses and interpretation of trends in FSW program outputs. Sex industry functioning was measured in terms of number of FSW continuing to work, number of “entertainment” area remaining open for business, number of clients reported by FSW, and number of health facilities and mobile clinics continuing to offer HIV testing services. Trends in FSW program outputs were assessed in terms of monthly numbers of FSW contacted via community outreach mechanisms, condoms distributed to FSW, FSW tested for HIV, as well as 3-month treatment retention rates among FSW that had initiated anti-retroviral therapy (ART). We assessed the statistical significance of magnitude of disruptions in the environment for commercial sex services, community outreach services for FSW, and HIV-related service delivery at health facilities via paired t-tests, which are appropriate for repeated measures data for districts that were used in the study [25].

## Results

By Southeast Asia country standards, Indonesia has been relatively hard hit by COVID-19. The first case was confirmed in early March. As of the end of July 2020, the reported number of confirmed coronavirus cases had reached 108,376 with 5131 deaths [23]. Due in part to limitations in testing capacity, the Government of Indonesia (GoI) was slow to respond to COVID-19. Policy responses included a “Work at home, study at home, pray at home” mandate, travel restrictions that limited mobility to/from Jakarta during and after the Muslim fasting month, suspension of most domestic airline flights, and mobilization of testing capacity to include all laboratories nationally that met designated standards. Gradual “re-opening” was begun in most provinces in June and July 2020.

**Fig. 1:**
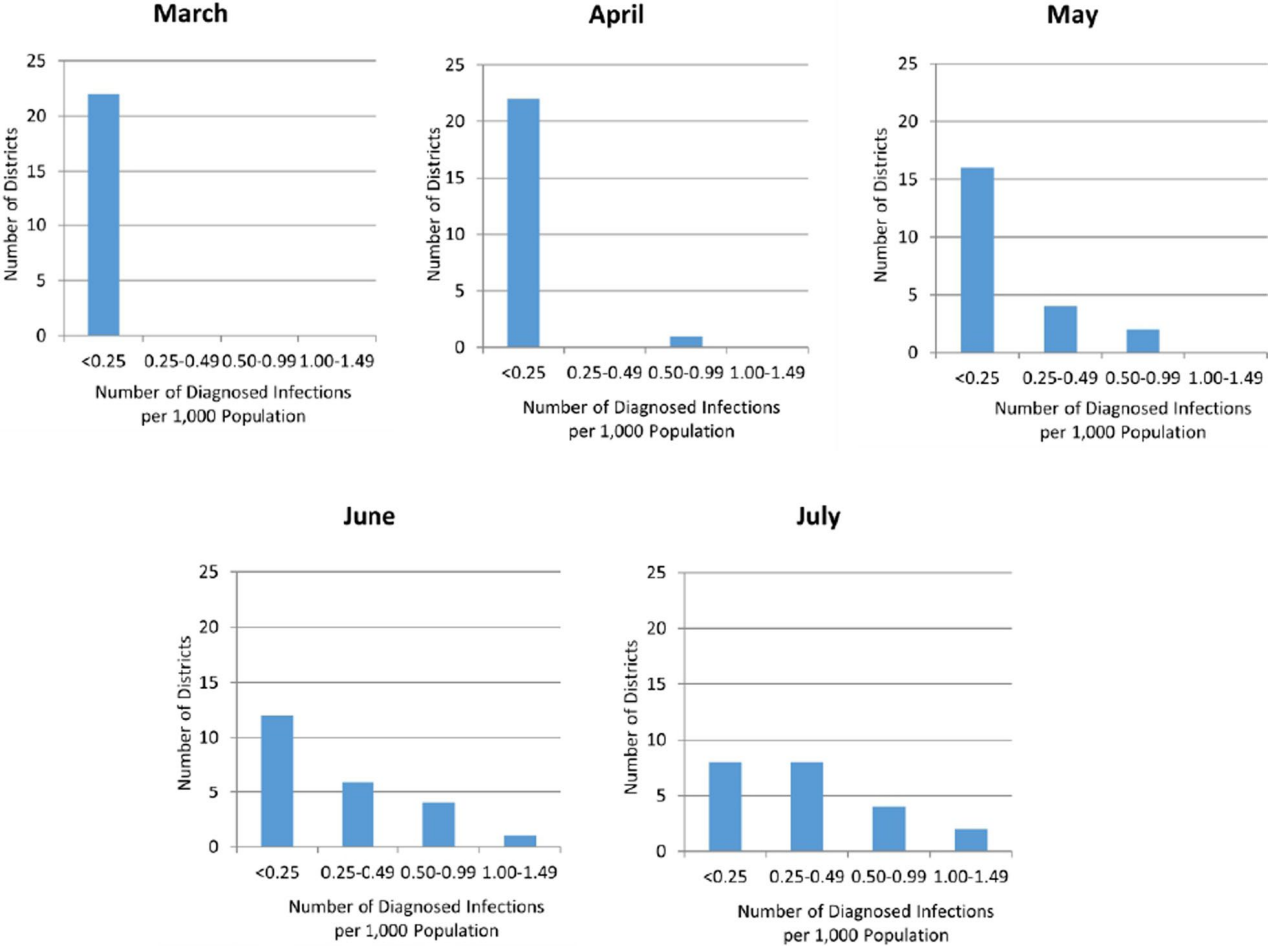
Monthly COVID-19 infection rates per 1000 population in study districts

Consistent with global experience, the timing of epidemic onset (detected at least) and effectiveness of containment varied geographically. The evolution of COVID-19 in the 23 study districts through July 2020 is summarized in Fig. 1. The figure displays the distribution of districts by infection rates (number of COVID-19 infections diagnosed infections per 1000 population) gleaned from provincial and district websites and other official communication portals (see Data links in the “Appendix” section). As may be observed, from a few “hotspot” districts in April (possibly associated with higher levels of testing), COVID-19 infection rates in study districts increased rather substantially through the month of July. An AVOVA test for trend was significant at *p* < 0.001.

**Fig. 2:**
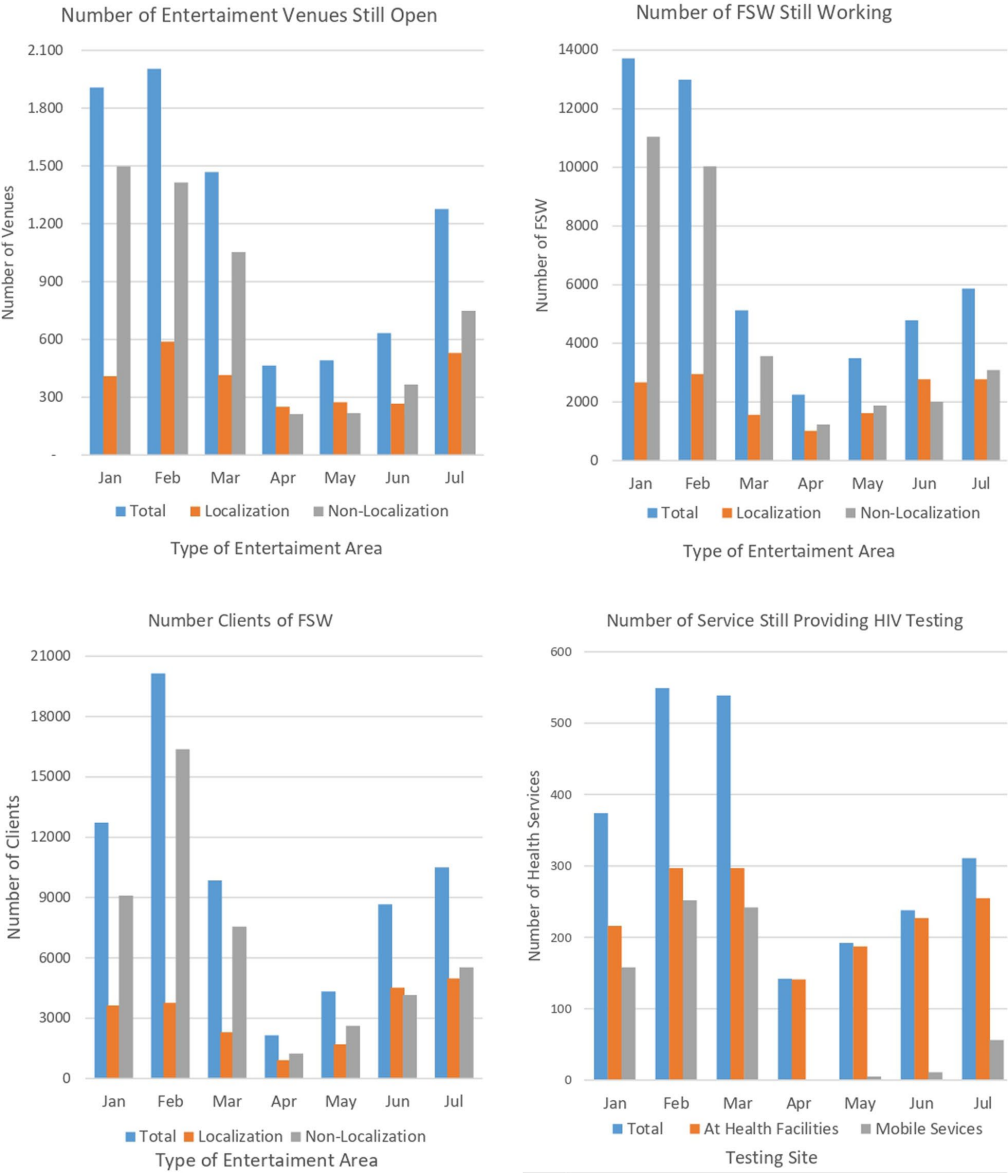
Trends in commercial sex activity and HIV testing service availability in 23 districts, January–July 2020

Figure 2 documents trends in levels of commercial sex activity and the availability of HIV testing services in the study 23 districts from January to July 2020 derived from monthly district scans. The level of commercial sex “hotspots” still functioning and numbers of both FSW and clients begin declining in March when travel restrictions and bans on large gatherings and “non-essential” businesses went into effect and fell to very low levels in April and May before rebounding in June and July. Declines in commercial sex in “non-Localization” areas were larger and recovery slower than in “Localization” areas (the term “Localization” refers to areas of cities that are quasi-legal and are managed as “entertainment” areas by local stakeholders). Availability of HIV health facility-based testing services was slower to be affected but fell precipitously in April before rebounding in June and July. Mobile clinic HIV testing services ceased in April and had only rebounded to about one-fifth of the February level by July.

The magnitude of reductions in commercial sex activities and HIV testing services measured as percent declines from the high point in the months of January–March 2020 to the low point in April–July for the 23 districts in the aggregate are presented in Table 1.

**Table 1:** District-level changes in commercial sex activity and HIV testing service availability, January–July 2020

Indicator	Highest number		Lowest number		% Reduction		
	High	Month	Low	Month	Aggregate*	Largest	Smallest
No. Localization areas still open	589	February	251	April	57	100	0
No. FSW still working—Localization Areas	2955	February	1015	April	66	100	35
No. client transactions—Localization	3760	February	906	April	76	100	14
No. Non-Localization areas still open	1415	February	213	April	86	100	30
No. FSW still working—Non-localization	11,042	January	1236	April	89	100	6
No. client transactions—Non-localization	16,382	February	1244	April	92	100	52
No. health facilities providing HIV testing	297	February	141	April	53	100	20
No. mobile clinics providing HIV testing	252	February	0	April	100	100	0

*All magnitudes of decline were statistically significant at the *p* < .01 level or greater in paired t-tests for repeated measures data

Also shown in the table are the largest and smallest district-level reductions in the indicators considered. In the aggregate, the three commercial sex activity indicators declined between 57 and 76% in Localization areas and between 86 and 92% in non-Localization areas. Some districts were literally shut down in terms of commercial sex activity, while in others the effects were nominal or greatly muted. The larger declines in numbers of entertainment areas remaining open and in numbers of female sex workers still working in Localization versus non-Localization areas was statistically significant (*p* < 0.05). The more muted impact in Localization versus non-Localization areas is likely due to the greater financial stake of local governments in the former and the desire to minimize economic fallout from COVID-19 mitigation actions. HIV testing at health facilities in the aggregate fell 53% between February and April and mobile clinic testing by 100%. Like commercial sex activity, the magnitude of effects varied by district from complete shutdown to nominal disruption.

**Fig. 3:**
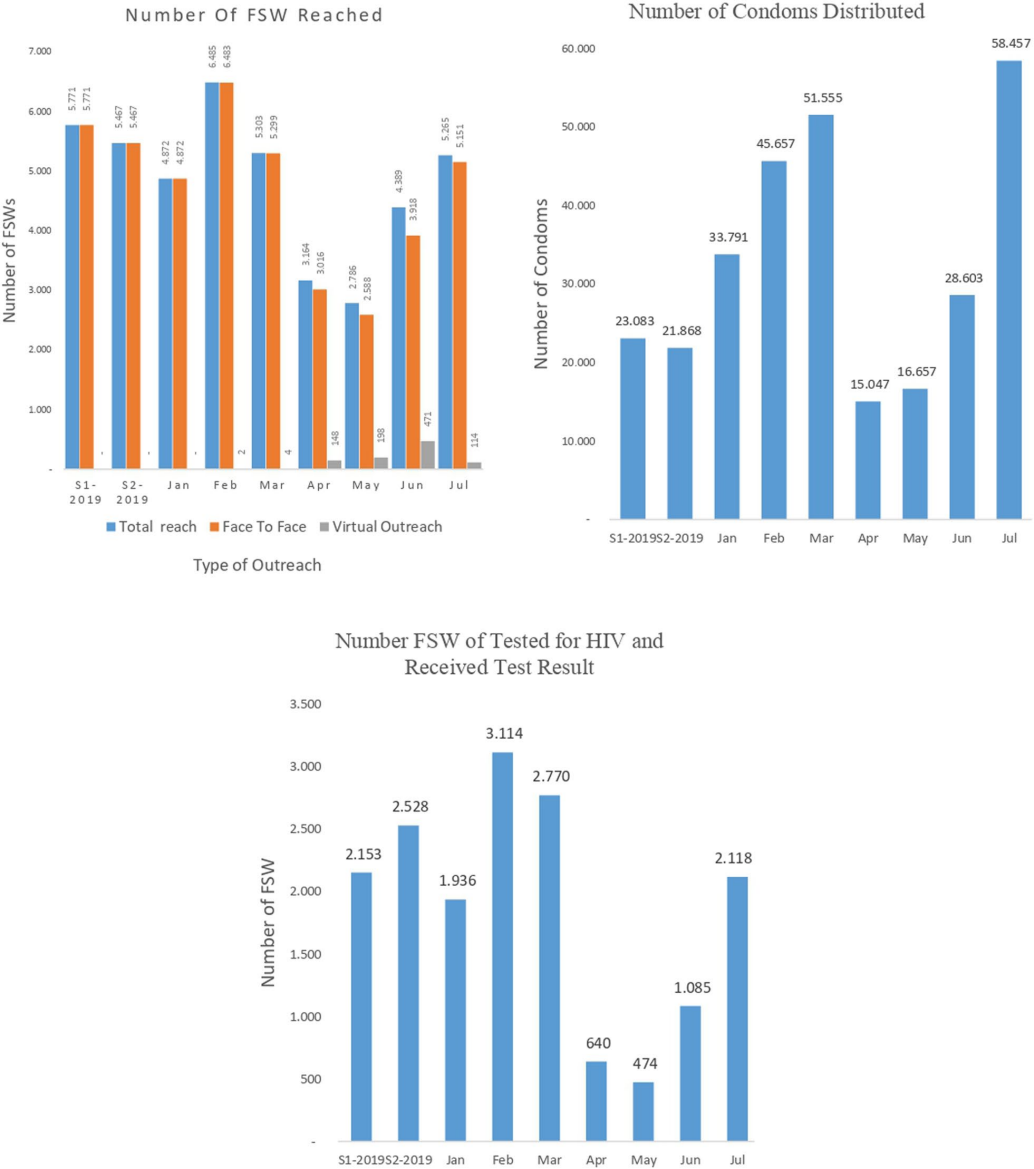
Monthly community outreach and HIV testing result for FSW in 23 districts, January–July 2020

How have these developments impacted the provision and use of HIV prevention and testing services by FSW? Figure 3 presents data on trends in FSW program outputs in the 23 study districts during January–July 2020. Also included in the data displays are monthly averages for January–June and July–December 2019 to provide a pre-COVID-19 benchmark for interpreting the 2020 monthly data. Outreach contact levels during **January–March** were roughly comparable to monthly averages during 2019. These fell dramatically during the months of **April and May**, undoubtedly at least in part reflecting the declines in the number of FSW that continued to work during those months. However, outreach contracts rebounded strongly in **June and July**. Similar trends are observed in condom distribution, HIV testing and, as is shown in Table 2, numbers of HIV cases detected and treatment initiation rates. The March-to-May declines in all three indicators in the table were statistically significant (*p* < 0.05 or better). It is to be noted that the testing-to-treatment “linkage” rates during April–July (83%) approximated the average monthly rate during the last half of 2019 or in January–March 2020 prior to the outbreak (both 76%), perhaps the result of FSW having delayed treatment initiation in earlier months.

**Table 2:** HIV case finding and treatment initiation rates among FSW in 23 districts, by month, January–July 2020

Service output	2019		2020						
	1st Semester	2nd Semester	Jan	Feb	Mar	Apr	May	Jun	Jul
Number HIV cases detected	31	34	20	48	41	16	8	19	34
Number initiating treatment	16	26	21	32	30	16	2	17	29
% Initiating treatment	52	76	105	67	73	100	25	89	85

Table 3 quantifies the magnitudes of contraction of program output indicators from the high point in the months of **January–March** to the low point in **April–July** along with the extremes of district responses. The low point for most indicators occurred in the month of May with high-low gaps ranging from 57 to 94%. Numbers of FSW reached via community outreach and numbers of condoms distributed were the least affected in the aggregate (*p* < 0.05), although the range of district-level responses on both indicators was extreme (11–91% for numbers of FSW reached and 0–100% for condoms distributed).

**Table 3:** District-level changes in FSW HIV program outputs, January–July 2020

Indicator	Highest number		Lowest number		% Reduction		
	High	Month	Low	Month	Aggregate*	Largest	Smallest
No. FSW reached	6485	February	2786	May	57	91	11
No. FSW tested for HIV	3114	February	474	May	85	100	76
No. Condoms distributed	38,885	February	16,375	May	62	100	0
No. HIV + cases detected	48	February	8	May	83	100	67
No. HIV + FSW initiating ARV	32	March	2	May	94	100	89

*All magnitudes of decline were statistically significant at the *p* < .001 level or greater in paired t-tests for repeated measures data

**Fig. 4:**
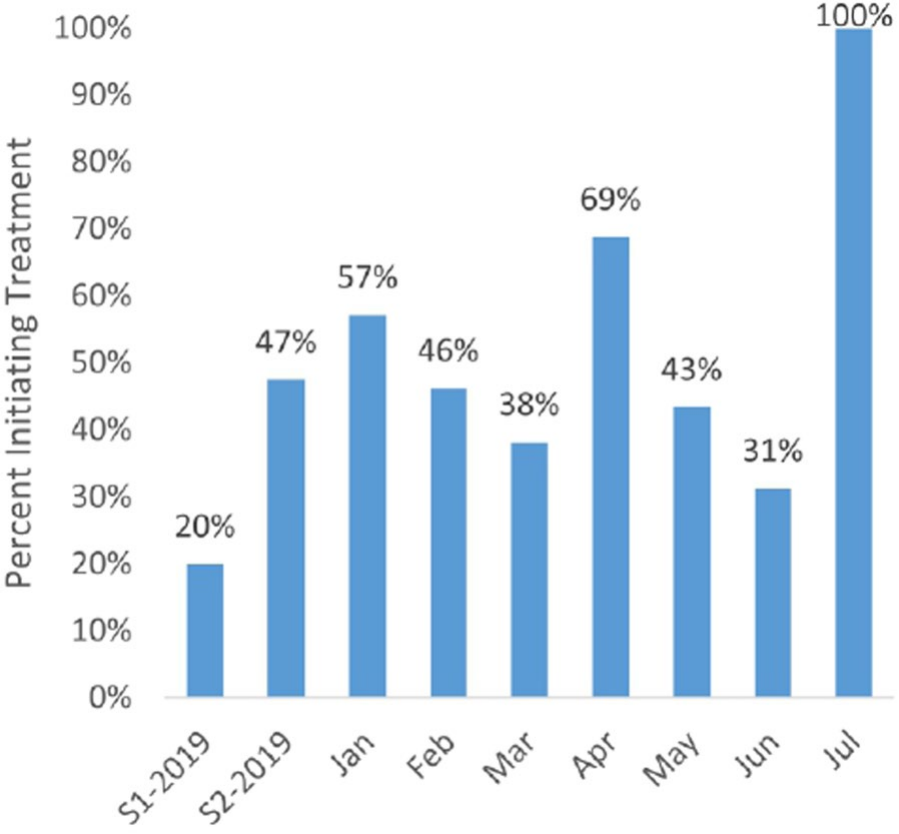
Proportion of FSW initiating treatment in the prior 3 months who were still on treatment, by month, January–July 2020

Figure 4 displays trend data on the proportion of FSW who had started ART 2 months prior to the reference month that were still on treatment at the end of the reference month. These data indicate a need for improved treatment retention rates but show no clear relationship with the onset of COVID-19. The July figure should be viewed with caution as it is based upon a very small number of FSW initiating treatment 2 months prior.

In April 2020, a community randomized trial of community HIV screening using an oral fluid-based rapid test was initiated with 15 of the 23 cities/districts being randomly assigned to receive the intervention and the other eight serving as controls. The study was planned well before the onset of COVID-19. Although it is still quite early in the study, the results through the end of July 2020 indicate significant demand for community HIV screening among FSW during a period of time of reduced HIV testing service availability at health facilities and via mobile clinics (Table 4). Parenthetically, although the numbers are small the community screening results are quite promising, with an overall 80% treatment initiation rate among FSW with confirmed reactive test results, substantially higher than the overall transition rates observed in Fig. 3. The proportion of reactive community tests that were confirmed at a health facility was lower (60%), which may have been influenced by the reduced availability of HIV testing services at health facilities during the period of observation.

**Table 4:** Community HIV screening, April–July 2020

Indicator	Month				Total
	Apr	May	Jun	Jul	
No. FSW tested for HIV	47	203	539	326	1115
No. reactive tests	1	4	9	11	25
Positivity rate	2.1	2	1.7	3.4	2.0
No. HIV + receiving confirmatory test	1	1	6	7	15
Pct. HIV + receiving confirmatory test	100	25	66.7	63.6	60
No. with confirmatory reactive results	1	1	6	7	15
Pct. with reactive confirmatory test	100	100	100	100	100
No. confirmed HIV + initiating ART	1	1	4	6	12
Pct. initiating treatment	100	100	66.7	85.7	80

## Discussion

Our findings suggest that COVID-19 has negatively affected HIV and AIDS combination prevention efforts for FSW in Indonesia, although the extent of collateral damage varied considerably by program component. HIV testing and linkage with treatment have seen the largest short-term effects, with numbers of FSW tested, HIV-positive cases found, and initiating treatment all falling by 83–94% between the months of February and May. The decline in HIV testing service availability was offset to some extent by the introduction of community testing in 15 of the 23 districts (see below for further discussion). Although absolute numbers of FSW initiating treatment had declined dramatically at the low point in May, the “transition” rate of treatment initiation given a HIV case finding was actually slightly higher during April–July (72.7%) than in January–March (67.9%).

No clear trend was observed in the proportions of FSW initiating ART that remained on treatment for two-plus months. Recent correspondence in the Lancet HIV [25] noted an emerging ART supply emergency in the country. While our data did not indicate a sustained fall-off in retention rates, we are unable to ascertain whether switching of treatment regimen or rationing of numbers of doses per month were necessary to attain the observed retention results.

Community-based primary prevention support services for FSW appear to have been less affected and to have recovered somewhat faster than HIV testing services notwithstanding the decline in numbers of active FSW. By July, the numbers of FSW reached via community outreach and condoms distributed to FSW had returned to or exceeded pre-COVID-19 levels. The smaller relative decline in numbers of FSW reached via outreach versus the estimated number of FSW still working might be explained by a higher proportion of active FSW seeking out community support than was the case during “normal” times. While condom distribution by CSOs had fully recovered by July, we were unable to assess pandemic effects on actual condom use.

It would thus appear that pandemic effects on the national HIV and AIDS response for FSW have been cushioned by continued community-level support by CSOs. In addition to providing an alternative to health facility-based and mobile clinic HIV testing via community HIV testing (in 15 districts at least), CSO staff have provided ongoing community-based information–education–communications and prevention commodity support and have facilitated HIV testing at health facilities (both initial and confirmatory), treatment initiation and treatment retention for the first 3 months of treatment. Such support is currently being provided in 96 (out of 514) districts. Where such support was not being provided, COVID-19 impact is likely to have been larger.

The preliminary results from the community HIV screening study are quite revealing. The positive response to community HIV testing might to some extent be merely a response to declining short-term availability of HIV testing services at health facilities and via mobile clinics. However, the fact that 72% of FSW availing of community screening as of the end of July 2020 were first-time testers suggests that community testing is reaching a segment of the FSW population that was not being reached by existing testing services. This is consistent with global evidence regarding the comparative advantages of community- versus health facility-based testing [26–28]. The fact that 40% of FSW testing positive in community tests failed to go to a health facility for a confirmatory test is matter of concern, especially since a confirmatory test at a health facility is required for HIV-positive persons to be eligible for government-funded treatment services. To the extent that socially marginalized HIV “key populations” are reluctant to avail of government testing and treatment services as was also observed in a recent study among MSM is three Indonesian cities [29], the impact of community testing will be muted. The study findings serve as a reminder that COVID-19 responses do not negate the need to address stigma and discrimination toward socially marginalized sub-populations [30]. The preliminary community testing findings support recent calls for expedited HIV differentiated service delivery [31], expanded use of alternative service delivery platforms such as “telemedicine” [32], and expanded community-based action both during and beyond the COVID-19 pandemic [33].

The COVID-19 pandemic has also produced a short- to medium-term humanitarian crisis for a sizeable proportion of FSW in the 23 study districts due to the loss of income. While commercial sex activity in “Localization” areas of appears to have rebounded, overall employment and activity levels remain far below pre-COVID-19 levels in the 23 districts in the aggregate. Most Indonesian FSW have limited education [34] and given the loss of jobs and continuing restrictions on informal sector activities due to COVID-19 prospects for finding alternative sources of livelihood do not appear promising in the short run. Most FSW in Indonesia “fall through the cracks” in the Government of Indonesia’s economic safety net provisions during the COVID-19 crisis due to (1) commercial sex being an informal sector activity that is not officially recognized and (2) regulations requiring individuals eligible for economic assistance to have local ID card—many FSW are not legally registered in the location where they work. The trajectory for further recovery will depend upon national and local success in containing COVID-19, which will enable (or not) authorities to further loosen business and social contact restrictions. As the GoI is struggling with COVID-19 containment there is limited visibility as to the likely trajectory of commercial sex industry recovery.

Several limitations of the study are acknowledged. First, we infer that changes in sex market functioning and HIV and AIDS programmatic responses were associated with COVID-19 based upon the relative timing of events, GoI policy records and statements, and reports from service delivery personnel in the field, but we are unable to demonstrate causality in any statistical sense. Second, the source of data for the study were GoI COVID-19 surveillance data and health facility and community service provider routine data. The former may have been affected by the relatively low levels of COVID-19 in some communities and Indonesia as a whole, while the latter sources of data are subject to under-and over-reporting that may have been exacerbated by the onset of COVID-19. However, the consistency of findings across the several sources of data used in the study suggest that while there may have been some measurement error in the various data series, the overall conclusions of the study remain valid.

## Conclusions

COVID-19 appears to have had a negative short-run impact on the livelihoods of FSW and the provision of HIV and AIDS-related services. The service delivery impact on FSW has been cushioned by the availability of community-based services and support. Medium- to longer-term impact will depend upon government success in containing COVID-19 and the rate of adoption of service delivery modalities that simultaneously address the needs of FSW and minimize risk of COVID-19 transmission.

## Data Availability

The data that support the findings of this study are available from UNFPA FSW program and study of randomized community trial on HIV self-testing among FSW in 23 districts, but restrictions apply to the availability of these data, which were used under license for the current study, and so are not publicly available. Data are however available from the authors upon reasonable request and with permission of UNFPA and researcher of the study.

## COVID-19 Data Links

http://covid19.sumutprov.go.id/

https://www.instagram.com/diskominfo_sumsel/?hl=en

https://www.instagram.com/dinkeslampung/

https://www.instagram.com/dinkes_provbanten/?hl=en

https://riwayat-file-covid-19-dki-jakarta-jakartagis.hub.arcgis.com

https://twitter.com/bogordinkes?lang=en

https://corona.bekasikota.go.id/

https://covid19.bandung.go.id/data

https://ccc-19.depok.go.id/

https://siagacorona.semarangkota.go.id/

https://www.instagram.com/dinkessurakarta/?hl=en

https://malangkota.go.id/

http://infocovid19.jatimprov.go.id/

https://www.instagram.com/denpasarkota/

https://infocorona.makassar.go.id/

https://dinkes.papuabaratprov.go.id/

https://twitter.com/pemprovpapua?lang=en

## Abbreviations

AIDS: Acquired Immune Deficiency Syndrome
ART: Antiretroviral therapy
COVID-19: Corona Virus Disease 2019
CSO: Civil society organization
FSW: Female sex workers
GBV: Gender-based violence
GFATM: Global Fund for AIDS, TB and Malaria
GoI: Government of Indonesia
HIV: Human immunodeficiency virus
IUs: Implementing Units
LGBTI: Lesbian, Gay, Bisexual, Transgender, Intersex
OPSI: *Organisasi Perubahan Sosial Indonesia*/Indonesian National Association of Sex Workers
PLHIV: People living with HIV and AIDS
TB: Tuberkulosis
UNAIDS: United Nations Programme on HIV/AIDS
UNFPA: United Nations Population Fund
WHO: World Health Organization
